# A State-of-the-Art Review of Intra-Operative Imaging Modalities Used to Quality Assure Endovascular Aneurysm Repair

**DOI:** 10.3390/jcm12093167

**Published:** 2023-04-28

**Authors:** Petra Z. Bachrati, Guglielmo La Torre, Mohammed M. Chowdhury, Samuel J. Healy, Aminder A. Singh, Jonathan R. Boyle

**Affiliations:** 1 Cambridge Vascular Unit, Cambridge University Hospitals NHS Foundation Trust, Cambridge CB2 0QQ, UK; 2 School of Clinical Medicine, Cambridge University, Cambridge CB2 0SP, UK

**Keywords:** endovascular aneurysm repair, imaging, computerised tomography, digital subtraction angiography, fusion, ultrasound

## Abstract

Endovascular aortic aneurysm repair (EVAR) is the preferred method for elective abdominal aortic aneurysm (AAA) repair. However, the success of this technique depends greatly on the technologies available. Intra-operative imaging is essential but can come with limitations. More complex interventions lead to longer operating times, fluoroscopy times, and greater contrast doses. A number of intra-operative imaging modalities to quality assure the success of EVAR have been developed. A systematic literature search was performed with separate searches conducted for each imaging modality in the study: computed tomography (CT), digital subtraction angiography (DSA), fusion, ultrasound, intra-operative positioning system (IOPS), and non-contrast imaging. CT was effective at detecting complications but commonly resulted in increased radiation and contrast dose. The effectiveness of DSA can be increased, and radiation exposure reduced, through the use of adjunctive technologies. We found that 2D-3D fusion was non-inferior to 3D-3D and led to reduced radiation and contrast dose. Non-contrast imaging occasionally led to higher doses of radiation. Ultrasound was particularly effective in the detection of type II endoleaks with reduced radiation and contrast use but was often operator dependent. Unfortunately, no papers made it past full text screening for IOPS. All of the imaging techniques discussed have advantages and disadvantages, and clinical context is relevant to guide imaging choice. Fusion and ultrasound in particular show promise for the future.

## 1. Introduction

Minimally invasive aortic surgery has been practised since the mid-1980s [1]. Since its inception, outcomes from endovascular aneurysm repair (EVAR) have been compared to open aortic repair. EVAR Trial 1, DREAM, and OVER did not demonstrate the mortality benefit of EVAR over open surgery beyond 30 days [2,3,4,5]. Despite new evidence regarding suboptimal long-term outcomes of decreasing survival benefit over time and almost double the reintervention rate compared to open aneurysm repair, it remains an attractive surgical intervention in those patients who are not physiologically capable to withstand open surgery [6,7]. What can be achieved with endovascular surgery, however, in large part depends on the technology used and accurate device deployment at the time of intervention. More complex repairs require longer fluoroscopy times, higher contrast doses, and greater exposure to ionising radiation to patients and interventionalists [8]. Imaging is fundamental to the correct approach and performance of EVAR and is categorised as pre-operative, peri-operative and post-operative. Pre-operatively, computed tomography angiogram (CTA) imaging plays a crucial role in the diagnosis and planning of the endovascular procedure. Intra-operatively, fluoroscopy and novel fusion imaging techniques aid the accurate deployment of stent grafts. Post-operatively, CTA and duplex imaging in surveillance allow for the detection of complications, with a particular focus on endoleaks [9]. The ESVS guidelines discuss the use of digital subtraction angiography (DSA) and intravascular ultrasound (IVUS) in the perioperative setting but conclude that these techniques are currently not widely available, difficult to perform, and add additional procedure time. These guidelines highlighted angiographic CT as a promising technique for the detection of complications, albeit with limited evidence presently [10]. Further, the introduction of fusion imaging has promised to revolutionise the EVAR technique by allowing a wider scope of intervention.

This review will aim to evaluate the role of CT, DSA, fusion, ultrasound, and non-contrast imaging for the detection of complications, radiation exposure, and contrast usage intra-operatively in EVAR.

## 2. Materials and Methods

**Figure 1:**
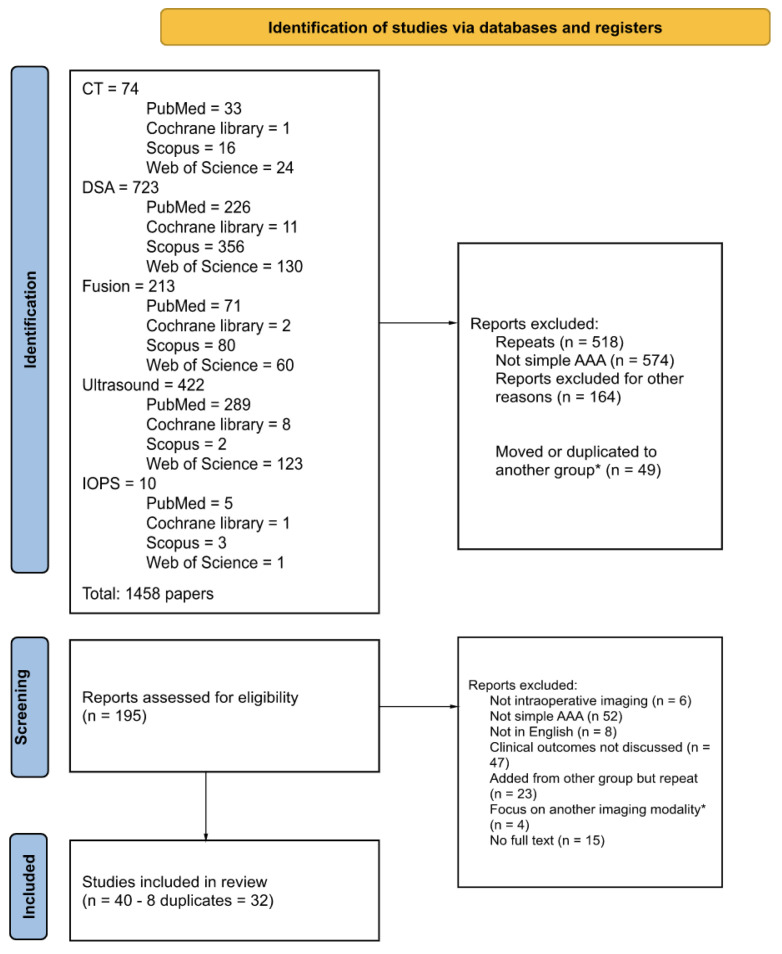
PRISMA Diagram. * those focusing on another imaging modality were moved or rarely duplicated to the relevant group and screened.

A systematic literature search of PubMed, Scopus, Web of Science, and Cochrane Library was performed on 7 February 2022. Separate searches (Supplementary Table S1) were conducted for each of the imaging modalities in the present study; CT, DSA, fusion, ultrasound, intra-operative positioning system (IOPS), and non-contrast imaging. Title, abstract screening, and full text review were conducted independently by authors PZB and SJH. A third independent author verified findings (GLT). Data extraction was carried out by PZB and SJH, following a predetermined standardised method. The data collected included author, year of publication, DOI, image modality, type of endovascular intervention, study type, sample sizes, sex of participants, and information regarding detection of complications, radiation dose, and use of contrast. Following inclusion and exclusion criteria (Table 1), a total number of 32 studies were included in the review (Figure 1 PRISMA Diagram). Relevant complications of EVAR were defined predominately as endoleaks but included stent kinking or compression, thrombosis, or renal function decline. Risk of bias was calculated using the Newcastle-Ottawa Scale [11].

## 3. Results

The studies considered in this review were heterogeneous. Patient populations were pooled according to the imaging modality and study type where possible. Where this was not possible, the results were reported on a study-by-study basis. A summary of the included studies can be found in Table 2.

### 3.1. Computerised Tomography

Intra-operative CT imaging during EVAR utilises an intravenous contrast agent, and there are different techniques in which images can be acquired. The recently developed cone beam CT (CBCT or dynaCT) involves converging beams and rotational flat panel detectors that allow accurate CT-like three-dimensional images to be produced. Multidetector CT (MDCT) uses multiple detectors to generate three-dimensional images [17].

#### 3.1.1. Detection of Endoleaks 

CT imaging allows the increased detection of endoleaks and technical complications intra-operatively and aids stent graft deployment. Törnqvist et al. [43] compared completion angiography and CBCT and suggested the need for multiple projections to compensate for the two-dimensional approach of angiography results in increased operating time and contrast use that may be offset using three-dimensional techniques such as CBCT. They concluded that CBCT is more effective at detecting stent graft compression and kinks, but angiography is better at detecting endoleaks, although the majority of these were type 2, which required no intervention. Schulz et al. [36] compared contrast-enhanced CBCT (ceCBCT) to completion DSA and post-operative CTA. All endoleaks found on DSA and CTA were also found on ceCBCT, but ceCBCT also detected intraluminal thrombus and limb stenoses, prompting intra-operative intervention in some cases. The authors suggest that completely replacing DSA and CTA with ceCBCT would result in a 38.8% reduction in the overall contrast used on the patient. Biasi et al. [12] compared dynaCT to completion DSA and found that 3.8% of the DSA group had a potentially preventable early re-intervention due to technical complications that were not identified during completion DSA. Patients undergoing an early reintervention for a secondary procedure had a statistically significantly higher mortality rate (14.3% vs. 3.3%). Their study showed no technical problems identified in pre-discharge surveillance imaging after dynaCT completion imaging, which was not the case with the completion DSA cohort, suggesting the superiority of dynaCT in assessing technical success. In contrast to previous studies, they did not find a statistically significant difference in contrast load between the DSA and the dynaCT groups, although there was an increase in radiation dose to the patient. Dijkstra et al. [17] evaluated patients undergoing fenestrated EVAR (FEVAR) and compared two protocols of imaging: pre-deployment CBCT fused with pre-operative multidetector CT (MDCT) to guide stent graft placement and post-deployment CBCT to assess technical success. For the post-deployment CBCT group, eight endoleaks were detected; all type I and type III endoleaks were resolved with adjunctive procedures, whilst the two type II endoleaks were left untreated. No endoleaks were found on pre-discharge MDCT that were not seen on CBCT. The contrast dose was significantly less for CBCT than MDCT, as was the radiation exposure.

#### 3.1.2. Radiation Exposure 

CT is associated with greater radiation exposure than DSA or other imaging techniques. Steuwe et al. [40] compared radiation exposure between intra-operative CBCT and post-operative follow-up MDCT and found that ceCBCT resulted in an average effective dose that was around 90–125% higher than a single venous phase MDCT image covering the same body area. However, with the actual MDCT protocol that was required to image the patients, intra-operative CBCT reduced the average effective dose by 60–65%. This difference was replicated in their phantom studies. 

CBCT is found by these studies to be superior when compared to angiography and DSA in detecting technical complications, particularly better or non-inferior at detecting endoleaks. As a result, CBCT may allow intra-operative correction of endoleaks and graft kinks and reduce the rates of post-operative complications and subsequent secondary interventions. The increased contrast doses and radiation doses compared to DSA and angiography may be offset by the increased efficiency of CBCT, reducing the need for further imaging and therefore the total contrast and radiation dose of the patient. 

### 3.2. Digital Subtraction Angiography 

Digital subtraction angiography (DSA) uses a pre-contrast ‘mask’ image, which is then digitally subtracted from an image taken after contrast injection. The requirement of multiple images to be taken to obtain one image often results in higher radiation doses when compared to simple fluoroscopy [44].

#### 3.2.1. Detection of Endoleaks 

Faries et al. [18] compared standard completion angiograms with a modified angiographic protocol, which involved DSA continuously for 60 s after injection of 20 mL of iodinated contrast media in the pararenal aorta and within the graft. With the standard protocol, type II endoleaks were detected in 6% of patients vs. 41% with the modified protocol (*p* < 0.001). However, during follow-up, no significant difference was noted in the incidence of type II endoleaks.

#### 3.2.2. Radiation Exposure 

Timaran et al. [42] compared the radiation doses between standard magnification and dual fluoroscopy with live-image digital zooming during fenestrated-branched EVAR (F/B-EVAR). Procedures performed with the dual fluoroscopy with live image digital zooming resulted in significantly lower median patient and theatre staff radiation doses compared to standard electronic magnification, with no difference in the technical success, procedure time, or fluoroscopy time of the procedures. de Ruiter et al. [16] compared fixed C-arm fluoroscopy with mobile C-arm fluoroscopy and the addition of image processing technology in the form of the Allura ClarityIQ technology. They found that for non-complex EVAR procedures, there was no significant difference in fluoroscopy time between the groups. However, there was a significant difference in total radiation exposure between the fixed and mobile C-arm groups, with the mobile C-arm having reduced radiation, which was replicated for complex EVAR procedures.

The studies included here primarily focused on modifications to DSA protocols to improve on the limitations of DSA. The addition of technological adjuncts can reduce the radiation dose, whilst the modification of contrast injection and fluoroscopy timing was able to provide more information about endoleaks. These are often the limitations of DSA that are improved upon by other imaging modalities.

### 3.3. Fusion Imaging 

Fusion imaging provides a patient-specific roadmap of blood vessels based on the fusion of intra-operative imaging with pre-operative imaging; this is most often a pre-operative CT angiogram. The intra-operative image may be DSA, fluoroscopy, or CBCT. Fusing the pre-operative CTA with intra-operative DSA or fluoroscopy provides a 2D-3D image, whereas fusion with intra-operative CBCT provides 3D-3D images [37]. This means that key operative landmarks can be continuously visualised throughout the operation without the need to continuously image, reducing patient exposure to excess radiation and contrast material. 

#### 3.3.1. Vascular Displacement after Stiff Wire 

Fusion imaging helps to provide accurate measurements of stiff wire localisation and resultant vascular displacement. In particular, Breininger et al. [13] showed its accuracy by manually segmenting 2D images and fusing them with preoperative 3D CTA. Further work by Lalys et al. [29] set out to quantify vascular displacement after stiff wire insertion via a pre-op 3D reconstruction and 2D intra-operative fluoroscopic imaging. Significant displacement was picked up by the fusion imaging, with a mean error of 4.1 ± 2.4 mm at the level of the renal arteries. Similarly, Maurel et al. [32] aimed to quantify vascular displacement with the fusion of pre-operative CTA and perioperative ce-CBCT with fluoroscopic guidance. This fusion imaging modality was able to pick up a median vascular displacement of the MA of 6.7 mm with reduced overall use of contrast. They also found a strong correlation between body mass index (BMI) and the amount of radiation used by the ceCBCT. Similarly, Jansen et al. [23], used pre-operative CTA and intra-operative ceCBCT. This fusion modality was able to detect an average displacement of target vessels, encompassing coeliac, SMA, and renal arteries of 7.8 mm. 

#### 3.3.2. Image Registration

Koutouzi et al. [28] compared automatic vs. manual (based on the L1-L2 position) 3D-3D imaging registration. Of the manually registered scans, 7/19 showed sufficient accuracy in the alignment of the renal arteries when this was based on the L1-L2 position for EVAR. The remaining error with 3D-3D registration showed the ongoing need for pre-deployment DSA. Neither 2D-3D nor 3D-3D fusion was shown to successfully completely replace intra-operative angiograms. Panuccio et al. [34] also investigated the role of a fully automated co-registration fusion imaging engine of preoperative CTA and intra-operative fluoroscopy, which was successful in 92% of cases. Stangenberg et al. [39] showed that the utilisation of correct, up-to-date software decreased the necessary radiation dose, fluoroscopy time, and contrast agent dose.

#### 3.3.3. 2D-3D vs. 3D-3D

Schulz et al. [37] compared 2D-3D (fluoroscopy and CBCT) vs. 3D-3D (CBCT and CBCT) fusion imaging. They showed the non-inferiority of 2D-3D compared to 3D-3D, but it had advantages in terms of radiation exposure and timeframe. Dijkstra et al. [17] compared the outcomes of intra-operative CBCT-MDCT fusion imaging with post-procedural CBCT and pre-discharge MDCT in FEVAR surgery. Fusion imaging resulted in overall lower contrast and skin doses. Schwein et al. [38] assessed the role of CTA-fluoroscopy fusion imaging in FEVAR. In total, 83% of blood vessels were successfully cannulated with the aid of fusion imaging alone without need for dedicated angiograms. These results show that 2D-3D fusion imaging may be precise enough to be more widely implemented but also offer lower radiation exposure and lower operative time.

#### 3.3.4. Radiation Exposure 

Tenorio et al. [41] found significant decreases in operator radiation exposure and effective dose in F-BEVAR with the use of fusion imaging. Furthermore, patients that had fusion imaging had significantly lower mortality (3% lower relative risk), incidences of major adverse events (24% lower relative risk), and need for secondary interventions (6% lower relative risk) at 30 days. McNally et al. [33] focussed on patients undergoing FEVAR or BEVAR. Fusion imaging provided a significant decrease in radiation exposure, fluoroscopy time, and contrast usage. The results were reproducible for three and four vessel stents. The estimated blood loss also decreased significantly. Results found by Rolls et al. [35] confirmed that fusion imaging significantly lowered exposure to ionising radiation and procedure time during FEVAR. Finally, Hertault et al. [22] confirmed that fusion imaging with a good collimator technique allows the achievement of very low radiation exposure doses.

#### 3.3.5. Reduction of Iodinated Contrast 

Kobeiter et al. [26] first reported the feasibility of CTA and low-dose CBCT fusion imaging without injection of iodinated contrast in FEVAR. Gallitto et al. [19] investigated the role of carbon dioxide angiography imaging vs. iodinated contrast imaging in the overall reduction of injected contrast medium during FEVAR. Carbon dioxide angiography led to overall lower doses of injected contrast media and similar detection rates of type 1, 2, and 3 endoleaks. The median hospitalisation in the carbon dioxide angiography group was significantly lower. Kaladji et al. [24] also set out to investigate the safety and usefulness of performing EVAR without pre- or intra-operative contrast. Six patients were enrolled due to low eGFR (median 17.5 mL/min/1.73 m^2^). No intra-operative endoleak was noted on duplex scanning, and there were no changes in eGFR at 1 week or 1 month. The stent graft position was achieved satisfactorily.

### 3.4. Non-Contrast Imaging 

Non-contrast imaging encompasses various techniques of intra-operative imaging during EVAR that attempt to reduce the use of iodinated contrast media (ICM). These imaging techniques include carbon dioxide DSA (CO_2_-DSA), gadolinium-enhanced magnetic resonance angiography (MRA), and non-contrast CT. In CO_2_-DSA, gaseous CO_2_ is injected instead of contrast. The gas pushes away the blood column, allowing the visualisation of the affected vessel [45]. Gadolinium-enhanced MRA uses gadolinium, which is paramagnetic and can be detected through how it affects MR signals [46]. Both alternatives to ICM allow the enhancement of the target vessels during intra-operative imaging. In contrast, non-contrast CT simply does not use ICM. 

Bush et al. [14] compared patients with either renal dysfunction or an ICM allergy and compared them to those who received ICM. Intra-operatively, intravascular ultrasound (IVUS) was used to measure the aorta to ensure the correct deployment of stent grafts, and post-implantation aortography was used with gadolinium contrast media throughout the operation when necessary and at post-implantation to assess the successful deployment of the graft. There was no statistically significant increase in creatinine from baseline in any patient in the cohort. Chao et al. [15] analysed DSA with either iodinated contrast agents (ICA-DSA) or CO_2_-DSA supplemented with ICA-DSA when needed. The CO_2_-DSA group required longer fluoroscopy and operating times and experienced increased radiation exposure. Additionally, 13 of the 16 procedures required supplementation with ICA-DSA. There was no significant difference in the number of endoleaks detected or changes in renal function between groups. Both studies found their respective non-ICM-based imaging techniques to be technically successful in imaging during EVAR. 

Studies looking at non-contrast imaging techniques primarily focussed on reduction of iodinated contrast use. Chao et al. [15] quoted literature values of 2 to 16% incidence of renal deterioration associated with EVAR, indicating the importance of reducing renal insults, including the use of iodinated contrast. This highlights that contrast dose reduction should be considered not only in patients with existing renal impairment but in all patients undergoing EVAR. 

### 3.5. Ultrasound Imaging 

Ultrasound imaging uses soundwaves to obtain images and carries no radiation risk. Contrast enhanced ultrasound (CEUS) produces images based on the interaction between the ultrasound waves, oscillations, and resonance of microbubbles [27]. Intravascular ultrasound (IVUS) is another ultrasound-based imaging technique used to obtain imaging for EVAR. Here, a rotational catheter with ultrasound-emitting capabilities is inserted intraluminally, allowing 360-degree images inside the vessel to be obtained [47]. This allows for precise measurements of vessel diameter and vessel wall composition [14]. 

#### 3.5.1. Detection of Endoleaks 

Massoni et al. [31] compared intra-operative CEUS with completion DSA in the early detection of endoleaks. The two imaging modalities agreed in 65% of cases, but CEUS detected more endoleaks (25 vs. 11). In a further study in 2021, Massoni et al. [30] looked specifically at the use of CEUS in the detection of type Ia endoleaks. In two cases, a type Ia endoleak was missed by angiography but detected on CEUS, resulting in an adjunctive procedure. In case 3, DSA detected an endoleak thought to be a type Ia, however, CEUS identified it as a type II from a lumbar artery, and as a result, no adjunctive procedure was performed. Keschenau et al. [25] also looked at the efficacy of CEUS in endoleak detection in patients undergoing F-BEVAR or infrarenal EVAR. Similar to Massoni et al. [31] in 2019, they found CEUS to detect significantly more type II endoleaks than completion angiography. However, many of those seen on CEUS were not seen on the pre-discharge CTA. In a later stage of their study, Keschenau et al. [25] carried out CEUS examinations at the same time as the pre-discharge CTA and found that of the four patients examined (who had type II endoleaks on the post-implantation CEUS), three had slow-flowing type II endoleaks that were detected by CEUS but not by CTA. The authors argued the value of CEUS as an investigation that reduces both contrast and radiation dose, and is superior in detecting type II endoleaks; however, it remains unclear whether this has clinical relevance. 

#### 3.5.2. Stent Deployment 

Kopp et al. [27] used CEUS in their study to identify the proximal landing zone of the stent and to confirm complete aneurysm exclusion at the proximal and distal landing zone. They found CEUS to be successful in 14 out of 17 patients at identifying the infrarenal landing zone and successfully releasing the graft proximally. CEUS was also found to be successful at visualising the distal landing zone at the iliac bifurcation in 25 out of 28 iliac arteries. Additionally, CEUS identified significantly more endoleaks than angiography. Operative time was similar for both groups, but time for radiation exposure and contrast use was significantly lower in the CEUS group. In contrast, Gennai et al. [21] used IVUS as a post-deployment imaging technique to assess the success of BEVAR/FEVAR stent graft deployment in a retrospective study of 10 patients, with 33 target visceral vessels. IVUS was technically successful in all cases. An increase in the operating time with the addition of IVUS was noted; however, IVUS identified problems in 4 of the 33 bridging stents that were not identified by completion angiography. Given the 12% of bridging stent issues that were only detected by IVUS, the authors concluded that there was a benefit to using IVUS as an adjunctive imaging modality in B-FEVAR, especially given its lack of contrast use and radiation exposure.

#### 3.5.3. Measuring Stent Graft Size 

Garrett et al. [20] evaluated aorta measurements taken by CT and by IVUS. They also conducted these measurements on a phantom tube, comparing the CT, IVUS, and calliper measurements. No statistically significant difference was found between the imaging techniques for the phantom. However, 22 cases had a sufficient disagreement between the pre-operative CT and intra-operative IVUS to result in changing stent graft size. In four cases, patients were considered inappropriate for EVAR based on the CT measurements, but IVUS suggested they were candidates, and these patients had successful interventions. No type I endoleaks were noted. The authors argue that the flexible sheath of the IVUS behaves more like the stent graft and is thus able to show more accurately the fit of the proximal aortic neck. 

Ultrasound-based imaging techniques significantly reduce contrast and radiation dose and may be superior in the detection of endoleaks. However, the clinical relevance of these endoleaks is questioned in these studies. Both CEUS and IVUS had value in helping guide deployment of the stent graft, ensuring correct positioning both in standard infrarenal EVAR and more complex interventions with branches or fenestrations. 

### 3.6. Intra-Operative Positioning System 

Intra-operative positioning system (IOPS) is a novel endovascular navigation system that does not use radiation or contrast, instead using electromagnetic sensors to provide 3D roadmaps to guide intervention [48]. Unfortunately, no papers passed through the full text search stage for IOPS. 

### 3.7. Risk of Bias

The risk of bias was assessed using the Newcastle-Ottawa Scale, with a median score of 8 (IQR 6–8) for all included studies (Supplementary Table S2).

## 4. Discussion

This study reviewed intra-operative imaging techniques used to quality assure EVAR by identifying technical complications and endoleaks that can be corrected at the time of initial intervention to improve EVAR durability and reduce the need for reintervention. The overall data on these techniques are limited to a small series and are of poor quality.

Patients requiring aortic aneurysm repair often have multiple comorbidities. Pre-existing renal impairment or renal insults from intra-operative contrast use can complicate endovascular intervention. Further, following EVAR, surveillance imaging is required to assess for stent position, endoleaks, and other complications. This monitoring is primarily conducted with duplex ultrasound, but patients often receive a post-operative CT scan, which adds to the lifetime radiation burden. Safe patient care involves minimising renal insult and exposure to ionising radiation as far as possible. Operators and theatre staff are also regularly exposed to ionising radiation during these procedures. Where ALARA principles are not followed or where the use of protection is lax, there may be an increased risk of harm to the operator including cataracts, skin damage, or even cancer [49,50,51]. These risks can be stochastic, such as cancer where there is no threshold dose, or deterministic, such as cataracts, where there is a threshold dose above which effects are seen. Thus, efforts to reduce the use of ionising radiation during procedures are not just beneficial to the patient. If preventable complications are not detected intra-operatively, then regardless of efforts to reduce radiation exposure during the surgery, the patient will be further exposed during re-intervention.

This review found that CT was good for identification of complications, with CBCT most often used intra-operatively. Whilst contrast use and ionising radiation exposure tended towards higher than comparative imaging, authors argued this to be acceptable in the context of reducing the need for re-intervention. Studies involving DSA focussed on reduction of radiation exposure, and the different protocols studied succeeded in this. Fusion imaging found 2D-3D fusion to be non-inferior to 3D-3D. Fusion imaging was also found to be useful in measuring vascular displacement after the insertion of stiff guidewires. Ionising radiation exposure and contrast usage was lower for fusion imaging, to the benefit of both the patient and the operator. Studies looking at automatic registration found it to be variable, but it shows promise in the future with further developments. Data regarding fusion imaging, albeit heterogeneous, indicate its utility to reduce overall radiation dose to patients and staff. The latest European Society for Vascular Surgery (ESVS) guidelines on radiation safety are clear regarding the importance placed on the judicious use of ionising radiation, encouraging operators to follow the ALARA principle (as low as reasonably possible). The ALARA principle should be adhered to by using low-dose protocols and limiting fluoroscopy time and screening time [52]. To achieve this, the ESVS stresses the importance of utilising more advanced imaging techniques such as fusion imaging. Concurrently, our review found data supporting that fusion imaging may help achieve shorter operative time. We show that there are data available to support the wider implementation of fusion imaging to achieve ALARA radiation exposure. Unsurprisingly, non-contrast imaging provided lower doses of contrast to the patient, but depending on the imaging used, occasionally resulted in higher doses of radiation, for example, in CO_2_-DSA. Ultrasound was found to be effective, particularly in the detection of type II endoleaks. It frequently resulted in interventions with reduced radiation and contrast use, indicating it to be both safe and effective. However, it is not widely used and may be less effective in patients with higher BMIs. Additionally, it is highly operator-dependent and costly; thus, widespread use may be limited by this. IVUS was found to be useful in device kinks and endoleak detection but is costly due to disposable IVUS catheters and is not widely used. Furthermore, Fibre Optic RealShape (FORS) could show real promise in the future. This modality utilises fibre optic laser technology to enable real-time device visualisation. So far, this is not a widely available technique although it has been used with some degree of success both pre-clinically and in the clinical setting [53,54]. This novel technique also promises to further reduce exposure to ionising radiation.

## 5. Conclusions

This review provides an overall synopsis of the intra-operative imaging modalities used to quality assure endovascular aortic surgery. All of the imaging modalities discussed have advantages and disadvantages and can be of use if utilised appropriately. Recent advances in intra-operative fusion and ultrasound imaging modalities seem to be particularly promising for future developments and may reduce radiation doses to patients and operators.

## 6. Limitations

The overall data quality of this study is poor and heterogenous, making it difficult to draw robust conclusions. There are limited data on long-term outcomes after intra-operative CT fusion or IVUS that suggest these intra-operative imaging techniques reduce re-intervention rates or long-term aortic aneurysm rupture. This may be the case, but at present, there is insufficient evidence to support this claim.

## Figures and Tables

**Table 1:** Inclusion and Exclusion Criteria.

Inclusion Criteria	Exclusion Criteria
In English	Not in English
EVAR procedures	Not EVAR procedures
Intra-operative imaging	Involvement of iliac arteries in the aneurysm or not simple AAA (e.g., rupture or mycotic, etc.) Pre-operative or post-operative imaging only
Full text available	Clinical outcomes of imaging not discussed (e.g., purely technical papers, phantoms, etc.) Animal studies

**Table 2:** Summary of included studies.

Author	Year	Imaging Modality	Aneurysm Type	Study Type	n	Summary of Technical Success & Complications	Contrast Usage	Radiation Dose
Biasi et al. [12]	2009	DynaCT vs DSA	Infrarenal	Prospective	392	DynaCT found 5 (6.25%) complications not seen on completion DSA with 3.8% having immediate intervention	No difference	Increased
Breininger et al. [13]	2019	2D3D	Non-specified EVAR	Retrospective	19	Successfully reconstructs Iliac displacement after stiffwire insertion from a 2D image	-	-
Bush et al. [14]	2002	Gadolinium-enhanced MRA, non-contrast CT, gadolinium or CO2 aortography, and IVUS	Infrarenal	Retrospective	297	Non-contrast technically successful in all patients	Reduced	-
Chao et al. [15]	2007	CO2-DSA vs. ICA-DSA	Infrarenal	Retrospective	100	No significant difference in technical success between groups	Reduced	Increased
de Ruiter et al. [16]	2016	DSA (mobile C-arm vs fixed C-arm/allura vs. fixed c-arm /AlluraClarity)	Infrarenal, complex	Retrospective	85	Image processing technology adjuncts can significantly help to reduce radiation exposure	-	Reduced
Dijkstra et al. [17]	2011	CBCT and 3D-3D fusion	Complex	Retrospective	82	Fusion technical success non inferior. No additional endoleaks found on MDCT.	Reduced	Reduced
Faries et al. [18]	2003	Standard angiography vs modified protocol	Non-specified AAA	Retrospective	391	Modified protocol detected more type II endoleaks but there was no significant difference in incidence of type II endoleaks by follow-up.	-	-
Gallitto et al. [19]	2020	3D2D fusion with intraop CO2-DSA	Complex	Prospective	45	CO2 angiography results in better renal function preservation	No contrast use	Increased
Garret, Jr. et al. [20]	2003	CT vs. IVUS	Infrarenal	Retrospective	78	IVUS resulted in changing stent graft size (n = 22). 4 patients treated with EVAR using IVUS after preop CT suggesting unsuitable.	-	-
Gennai et al. [21]	2021	Fusion but vessel cannulation with IVUS	Complex	Retrospective	10	IVUS was technically successful in all cases, identifying problems in 12% of bridging stents that were not detected by completion angiography.	Reduced	Reduced
Hertault et al. [22]	2018	3D2D with strict ALARA	Infrarenal	Prospective	85	-	Reduced	Reduced
Jansen et al. [23]	2021	3D2D	Complex	Retrospective	20	-	-	-
Kaladji et al. [24]	2015	3D2D without contrast	Infrarenal, thoracic	Prospective	6	EVAR graft deployment	No contrast use	-
Keschenau et al. [25]	2020	CEUS vs. DSA	Infrarenal, complex	Prospective	21	CEUS detected significantly more type II endoleaks than DSA. But only 5 of the 16 still persisted on pre-discharge CTA.	Reduced	Reduced
Kobeiter et al. [26]	2011	3D2D without ICM for registration	Thoracic	Retrospective	1	TEVAR deployment	No contrast use	-
Kopp et al. [27]	2010	CEUS vs. DSA	Infrarenal	Prospective	37	CEUS was effective at identifying proximal (82.4%) and distal (89.3%) landing zones and identified more endoleaks than angiography.	Reduced	Reduced
Koutouzi et al. [28]	2016	3D3D registration and 2D3D overlay	Infrarenal	Prospective	19	EVAR deployment	Reduced	Reduced
Lalys et al. [29]	2019	3D2D fusion	Infrarenal	Prospective	50	Assessment of displacement	-	-
Massoni et al. [30]	2021	CEUS vs. DSA	Infrarenal	Prospective	3	In two cases type Ia endoleak was missed by angiography but detected by CEUS	-	-
Massoni et al. [31]	2019	CEUS vs. DSA	Infrarenal	Prospective	60	Postdeployment CEUS detected more endoleaks than DSA	-	-
Maurel et al. [32]	2014	3D3D	Infrarenal, complex	Prospective	20	Stiffwire insertion causes significant diplacement of main aortic branches	Reduced	Increased
McNally et al. [33]	2015	3D3D vs. fluoroscopy/DSA/IVUS	Complex	Retrospective	72	FEVAR deployment	Reduced	Reduced
Panuccio et al. [34]	2016	3D2D but with mathematical model	Infrarenal, complex	Prospective	25	Fully automated fusion imaging is possible although manual intervention may be needed in some cases	Reduced	Reduced
Rolls et al. [35]	2016	3D3D vs. standard fluoroscopic imaging	Complex	Prospective	42	Target vessel catheterisation and endoleak detection satisfactory. Fusion and team based approach reduced procedure time	-	Reduced
Schulz et al. [36]	2016	ceCBCT vs. cDSA	Infrarenal	Prospective	98	ceCBCT detected more endoleaks than CTA or DSA	Reduced	-
Schulz et al. [37]	2019	2D3D fusion vs. 3D3D fusion	Non-specified EVAR	Prospective	151	Fusion imaging is feasible, and non-inferior to 3D3D offering better radiation exposure and time demand	-	Reduced
Schwein et al. [38]	2018	3D-3D fusion and CTA-fluoroscopy	Complex	Retrospective	26	83% of ostia cannulated without angiogram	Reduced	Reduced
Stangenberg et al. [39]	2015	3D2D fusion using VesselNavigator	Infrarenal	Retrospective	75	Procedure time, fluoroscopy time and air kerma was lower with fusion	Reduced	Reduced
Steuwe et al. [40]	2016	CBCT vs MDCT	Infrarenal	Retrospective	66	CBCT reduces radiation dose compared to 3-phase MDCT required to assess technical success of EVAR	-	Reduced
Tenorio et al. [41]	2019	3D3D onlay CTA fusion and CBCT without digital zoom capability 2D3D onlay CTA fusion, high definition CBCT with subtraction capability and digital zoom.	Complex	Retrospective	386	Successful stent deployment and endoleak detection	Reduced	Reduced
Timaran et al. [42]	2021	Standard vs. dual fluoroscopy with live-image digital zooming	Complex	Prospective	151	No difference in technical success between the two groups	-	Reduced
Törnqvist et al. [43]	2015	CBCT vs. DSA	Infrarenal	Prospective	51	CBCT more effective at detecting stent graft compression and kinks. DSA detected more endoleaks than CBCT	-	-

## Data Availability

No new data were created or analyzed in this study. Data sharing is not applicable to this article.

## Supplementary Materials

The following supporting information can be downloaded at: https://www.mdpi.com/article/10.3390/jcm12093167/s1, Table S1: Search strategy; Table S2: Risk of bias.
