# Supplementary material for: A State-of-the-Art Review of Intra-Operative Imaging Modalities Used to Quality Assure Endovascular Aneurysm Repair
Source: J Clin Med. 2023 Apr 28;12(9):3167. doi: 10.3390/jcm12093167 (PMC10179131; doi:10.3390/jcm12093167)
Supplement: Supplementary file 1 [file jcm-12-03167-s001.zip › Supp Table 1.pdf]

Supplementary table 1 – search terms

- (Cone-beam CT OR angiographic CT OR C-arm CT OR Dyna-CT) AND intraoperative AND (EVAR OR endovascular aneurysm repair)
- (DSA OR digital subtraction angiography) AND intraoperative AND (endovascular OR EVAR)
- (CO2-angiography OR carbon dioxide-guided angiography OR CO2-EVAR OR non-contrast CT) AND intraoperative AND (endovascular OR EVAR)
- (IVUS OR intravascular ultrasound OR intravascular ultrasonography OR intraoperative contrast-enhanced ultrasonography OR CEUS OR contrast-enhanced ultrasound OR intraoperative duplex ultrasound) AND intraoperative AND (endovascular OR EVAR)
- (Fusion imaging AND intraoperative AND (endovascular OR EVAR)
- (IOPS OR intra-operative positioning system) AND intraoperative AND (endovascular OR EVAR)
