# Supplementary material for: A State-of-the-Art Review of Intra-Operative Imaging Modalities Used to Quality Assure Endovascular Aneurysm Repair
Source: J Clin Med. 2023 Apr 28;12(9):3167. doi: 10.3390/jcm12093167 (PMC10179131; doi:10.3390/jcm12093167)
Supplement: Supplementary file 1 [file jcm-12-03167-s001.zip › Supp Table 2.pdf]

Author	Selection	Comparability	Exposure/Outcome	Total
Biasi et al	*** ***	** **	*** ***	8
Breininger et al	***	**	***	8
Bush et al	***	*	**	6
Chao et al	***	**	***	8
De Ruiter et al	***	**	***	8
Dijkstra et al	***	**	***	8
Faries et al	**	*	**	5
Gallitto et al	***	**	***	8
Garrett et al	***	*	**	6
Gennai et al	***	*	**	6
Hertault et a	***	*	**	6
Jansen et al	***	**	***	8
Kaladij et al	***	*	**	6
Keschenau et al	***	**	***	8
Kobeiter et al	***	**	***	8
Kopp et al	***	**	***	8
Koutouzi et al	***	**	***	8
Lalys et al	***	**	***	8
Massoni et al	***	*	**	6
Massoni et al	***	**	***	8
Maurel et al	**	*	**	5
McNally et al	***	**	***	8
Panuccio et al	***	**	***	8
Rolls et al	***	*	**	6
Schulz et al	***	**	***	8
Schulz et al	***	**	***	8
Schwein et al	***	**	***	8
Stangenberg et al	***	**	***	8
Steuwe et al	**	*	**	5
Tenorio et al	***	**	***	8
Timaran et al	***	**	***	8
Tornqvist et al	***	*	**	6
